# Autocorrelated measurement processes and inference for ordinary differential equation models of biological systems

**DOI:** 10.1098/rsif.2022.0725

**Published:** 2023-02-22

**Authors:** Ben Lambert, Chon Lok Lei, Martin Robinson, Michael Clerx, Richard Creswell, Sanmitra Ghosh, Simon Tavener, David J. Gavaghan

**Affiliations:** ^1^ Department of Mathematics, University of Exeter, Exeter EX4 4PY, UK; ^2^ Faculty of Health Sciences, Institute of Translational Medicine, University of Macau, Macau, People’s Republic of China; ^3^ Department of Computer Science, University of Oxford, Oxford OX1 3QG, UK; ^4^ School of Mathematical Sciences, University of Nottingham, Nottingham NG7 2RD, UK; ^5^ MRC Biostatistics Unit, University of Cambridge, Cambridge CB2 0SR, UK; ^6^ Department of Mathematics, Colorado State University, Fort Collins, CO 80523, UK

**Keywords:** inference, Bayesian statistics, Fisher information, ordinary differential equations, autocorrelation, measurement error

## Abstract

Ordinary differential equation models are used to describe dynamic processes across biology. To perform likelihood-based parameter inference on these models, it is necessary to specify a statistical process representing the contribution of factors not explicitly included in the mathematical model. For this, independent Gaussian noise is commonly chosen, with its use so widespread that researchers typically provide no explicit justification for this choice. This noise model assumes ‘random’ latent factors affect the system in the ephemeral fashion resulting in unsystematic deviation of observables from their modelled counterparts. However, like the deterministically modelled parts of a system, these latent factors can have persistent effects on observables. Here, we use experimental data from dynamical systems drawn from cardiac physiology and electrochemistry to demonstrate that highly persistent differences between observations and modelled quantities can occur. Considering the case when persistent noise arises owing only to measurement imperfections, we use the Fisher information matrix to quantify how uncertainty in parameter estimates is artificially reduced when erroneously assuming independent noise. We present a workflow to diagnose persistent noise from model fits and describe how to remodel accounting for correlated errors.

## 1. Introduction

Ordinary differential equation (ODE) models are used throughout biology, typically to describe dynamic processes. Amidst a huge range of applications, ODEs are used to describe the transmission dynamics of infectious diseases [1]; they can represent the dynamics of enzyme-catalysed reactions [2] and can explain the formation of action potentials in neurons [3]. In ODE models, the evolution of a system depends only on its current state and a set of input parameters, which determine how individual components of the system interact. The parameters of ODE models in biological systems are typically not directly measurable and must be inferred from data. In this article, we consider the assumptions underpinning inference of parameters from biological data.

A typical ODE model for modelling a dynamic process may be written:

1.1 dxdt=h(t,x,θ), t∈(0,T]and x(0;θ)=x0,}

where $x(t;\theta )\in R^{n}$ is the state of the system; $\theta \in R^{m}$ are the parameters of the system; *t* denotes time; *h*(*t*, *x*, *θ*) can be a function of time, state and parameters; and $x_{0}\in R^{n}$ is the initial state.

We suppose that an ODE model is proposed to explain a dataset: ${\left\{\tilde{y}(t_{i})\right\}}_{i=1}^{N}$, where $\tilde{y}(t_{i})\in R^{l}$ and *l* ≤ *n*. By fitting the model to these data, an analyst hopes to recover estimates of the parameters, *θ*, which incorporate uncertainty. ODE models typically do not explain all variation within a dataset because they are approximations of the underlying processes, meant only to capture the most dominate characteristics of variation. Particularly in biology, the measurement of the system itself is also imperfect: measurement apparatus has a finite resolution and may provide indirect measures of the quantity of interest, and human errors may also contribute noise to observations. Because of these factors, a random error process is hypothesized to connect noisy observations with the ODE solution. This may be written as follows:

1.2 $$y(t_{i})=g(x(t_{i}))+\epsilon (t_{i}),$$

where $g\;:\;R^{n}\to R^{l}$ allows a measured quantity to be a function of the ODE solution. In equation (1.2), $\epsilon (t_{i})$ is a random variable that represents both the effects of model misspecification and measurement noise.

The canonical assumption for the error terms is that they represent independent and identically distributed (IID) draws from a normal distribution [4–9]: $\epsilon (t_{i})\overset{\mathrm{IID}}{∼}\;N(0,\sigma )$, where *σ* > 0 characterizes the width of this distribution. The IID normality assumption is so widespread that it is typically stated without justification.

The normality assumption may be justified on the basis of a central limit theorem if it is thought that a series of independent or weakly dependent random variables—representing different characteristics of measurement and misspecification processes—contribute additively to the overall errors; it may also be reasonable since the normal distribution emerges from a disparate range of processes representing measurement imperfections [10, ch. 7]. However, if there is strong correlation between these constituent parts, then a distribution with heavier tails, such as a Student-*t* distribution or a Huber distribution is more appropriate [11].

An IID normal distribution can also be justified by invoking the principle of maximum entropy [10,12]. This principle roughly states that a probability distribution representing the outcomes of a process of interest should be chosen to include as little possible information about a process subject to known constraints. If only the mean and variance of the outcomes of a process are known, and there is thought to be zero correlation between errors, then it can be shown that an IID normal distribution is the probability distribution that makes the fewest additional assumptions [10, ch. 7]. However, it is unclear how applicable this is to the error distribution for ODEs, since we typically know only that the mean of the error distribution is zero, and our empirical examples indicate that the independence assumption may be an unreasonable null hypothesis. In particular, if there is thought to be autocorrelation in the noise, then a multivariate normal over the errors is the distribution with maximum entropy.

**Figure 1.**
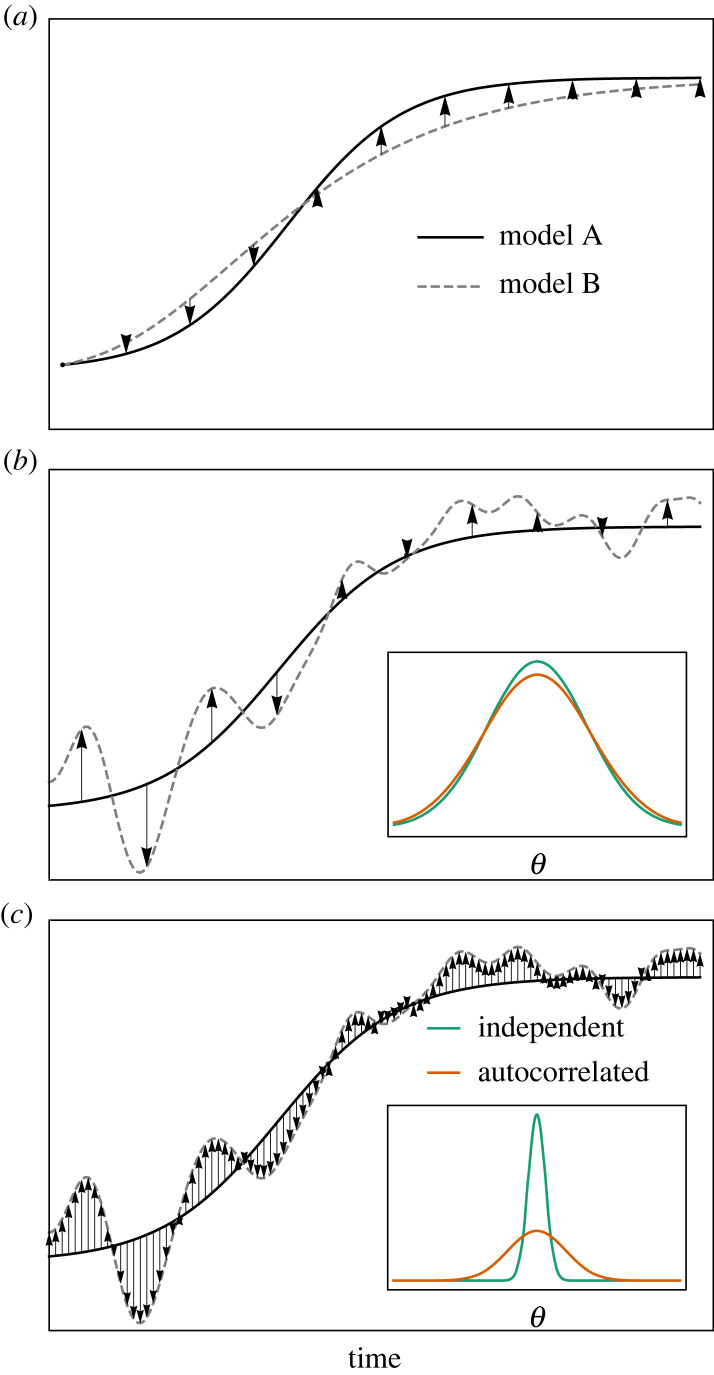
Causes of autocorrelated noise. (*a*) ODE model misspecification: shows how using a logistic model when, in fact, a Gompertz model is correct, results in autocorrelated noise; (*b*) and (*c*) show how an imperfect measurement process can lead to different characteristic residual noise processes: (*b*) the measurements are taken using a coarse grid; (*c*) the measurements are taken using a fine grid. In all cases, residuals are depicted by black arrows. The inset plots show representative posterior distributions under different assumptions about the measurement process.

There are two general causes of autocorrelation in the errors: misspecification of the model and poor temporal resolution of the measurement process [12]. In figure 1*a*, we illustrate how misspecifying an ODE model can lead to autocorrelated errors. This figure shows the outputs of two dynamic models as solid (model A) and dashed (model B) lines. We suppose that there is no measurement noise and that the data (arrow tips) is generated by model A. In attempting to fit these data, suppose model B is mistakenly chosen, and its best fitting line is as shown in figure 1*a*. There are manifold ways in which a model can be misspecified: the assumed functional form governing interactions between variables can be incorrect; important variables can be left out of the model entirely; a deterministic model may be used when a stochastic one is more appropriate; and so on. In this example, any of these issues could conceivably result in the differences between model A and model B, and, by choosing model B, this misspecification results in residuals (shown as arrows) exhibiting positive autocorrelation.

There is a huge literature devoted to accounting for model misspecification during inference [13–16], and this remains an active area of research. In this article, however, we focus only on the impact of assumptions around measurement noise, since, as we demonstrate, these can have dramatic effects on inference even in the absence of model misspecification. To exemplify how measurement process imperfections can lead to autocorrelation, suppose again that model A is the true model of nature, and that we (correctly) use it as part of our model of the data generating process. Also, suppose that the measuring apparatus is imperfect, producing noisy observations that may differ from the true underlying state and has finite temporal resolution, meaning it struggles to capture changes in output over shorter time scales. In figure 1*b*,*c*, we show the model solutions (solid lines) and the values that would be measured if using a very fine temporal gridding (dashed lines). A consequence of this smooth measurement process is that the more observations per unit time are taken, the greater the degree of autocorrelation in residuals. In figure 1*b*, we show coarse observations of the system of interest as indicated by the horizontal positioning of the vertical arrows. In this case, since observations are sufficiently separated in time, there is relatively low persistence in residuals. In figure 1*c*, we take more observations of the same process, which produces positively autocorrelated residuals.

Intuitively, when the measurement process is positively autocorrelated, each observation conveys less information about the system than when the observations are uncorrelated. So misrepresenting an autocorrelated error process with one assuming independence can lead to overly confident parameter estimates. This is a well-known result in regression modelling [17], and, since fitting ODE models to data is just nonlinear regression, these results should also apply to inference for these model types. We show this in the insets in figure 1*b*,*c*: here, the orange lines show (illustrative) posterior distributions resultant from modelling the measurement process correctly; the green lines show the distributions when modelling the measurements assuming independence amongst them. In figure 1*b*, where the measurements are widely spaced, there is little difference in the recovered posteriors owing to the limited autocorrelation. In figure 1*c*, failure to account for autocorrelation results in a posterior with too little variance.

We originally became interested in the impact of measurement autocorrelation on parameter estimation when attempting inference for a model of an electrochemistry experiment. We noticed that the estimates obtained were unrealistically precise when assuming an IID normal error model, and the errors were autocorrelated. This led us to consider how this phenomena might be more generally applicable and whether there were guiding principles of how the degree of overconfidence depends on measurement autocorrelation. Thus, in this article, we explore how measurement autocorrelation affects the precision of estimates. The previous work, in the context of modelling physical systems, has derived straightforward expressions for parameter uncertainty for a dataset consisting only of two time points with an accordingly simple error model [12]. Here, we consider a much more general setting where the models are nonlinear ODEs, which is typical in biological systems analysis, and the measurement process can be any one of a wide class of stochastic processes. We also account for the bias in the estimates of the standard deviation of the noise when fitting a model assuming IID Gaussian errors, which is important to ensure correct estimates of the degree of overconfidence. By using simulated data from ODE models, we demonstrate the validity of our analytical results. By using experimental data from cardiac physiology and electrochemistry, we show that highly persistent differences between observations and modelled quantities can occur. Whilst only illustrative, these results hint that overconfidence in parameter estimates may not be uncommon, particularly in systems with high-frequency measurements. In addition, we provide a workflow for diagnosing and accounting for autocorrelated errors when fitting an ODE model to data.

## 2. Effect of autocorrelated noise on parameter estimate uncertainty

In this section, we use mathematical analysis to evaluate the effect on parameter estimates of not accounting for autocorrelation when present. To do so, we first calculate ‘*true*’ parameter uncertainties obtained when specifying a persistent error model. We then calculate ‘*false*’ uncertainties obtained when assuming independent errors. To derive these quantities, we calculate the Fisher information matrix (FIM) in both circumstances. This analysis shows that uncertainty in parameter estimates is understated when (falsely) assuming independent errors, with the degree of overconfidence increasing along with the persistence of the true errors. We call the ratio of true parameter estimate variance to that estimated assuming independent errors the ‘*variance inflation ratio*’ (VIR).

In §2.1, we estimate the VIR for the mean parameter of a simple model with constant mean, when the actual error process is persistent and described by an autoregressive order-one (AR(1)) process. Calculating the VIR for the constant mean model is straightforward but provides a useful guide when examining more realistic cases. In §2.2, we consider a nonlinear ODE model with AR(1) measurement noise. In §2.3, we explore the consequences of more ephemeral autocorrelations by calculating the VIR for the constant mean model with moving average order-one (MA(1)) errors. Realistic noise processes are likely, in fact, to be combinations of persistent and transient correlated noise, and in §2.4, we give formulae for computation of VIRs in this, more general, case.

### 2.1. Constant mean model

In what follows, we assume a time series framework where, at time *t*, observed data, *x*(*t*), differs from its true constant value, *μ*, by an additive random component:

2.1 x(t)=μ+ϵ(t),

where ϵ(t) is a zero-mean error random process such that E[x(t)]=μ.

There are a number of ways that measurement errors may be autocorrelated, and, in this article, we consider a range. To begin, we consider AR(1) errors, in which there are persistent deviations between the observations and the true values of a process. This could occur, for instance, if a measurement apparatus responds slowly to changes in a system, meaning observations taken closer together are likely to be correlated owing to measurement imperfections. An AR(1) process can be represented mathematically by:

2.2 ϵ(t)=ρϵ(t−1)+ν(t),

where $\nu (t)\overset{\mathrm{IID}}{∼}\;N(0,\sigma )$, and −1 < *ρ* < 1 characterizes the degree of autocorrelation: positive values indicating positive autocorrelation; and similarly so for negative values.

We first derive the *true* (asymptotic) variance of the maximum likelihood estimator of *μ* when assuming an AR(1) error process in accordance with the true generating process. To do so, we use the log-likelihood to determine the diagonal element of the FIM corresponding to *μ* when we assume *ρ* is known. To write down the log-likelihood, we require an expression for *ν*(*t*) in terms of the observables and parameters of the system, which can be obtained by multiplying *x*(*t* − 1) given by equation (2.1) by *ρ* and subtracting it from *x*(*t*), resulting in *ν*(*t*) = *x*(*t*) − *ρx*(*t* − 1) − *μ*(1 − *ρ*). Since *ν*(*t*) is distributed as an independent Gaussian, the log-likelihood of the model for a sample of observations x(t) : ∀t∈[0,1,2,…,T] is given by:

2.3 $$L=-\frac{T}{2}\log2\pi -\frac{T}{2}\log\sigma^{2}-\frac{1}{2\sigma^{2}}\sum_{t=1}^{T}{\left(x(t)-\rho x(t-1)-\mu (1-\rho )\right)}^{2}.$$

Where, for simplicity, we have assumed that *ν*(0) = 0 is fixed and known—§3.2 describes an alternative likelihood that does not make this assumption.

The second derivative of equation (2.3) with respect to *μ* yields the relevant diagonal element of the FIM:

2.4 $$I_{\mu ,\mu }=-E\left[\frac{\partial^{2}L}{\partial \mu^{2}}\right]=\frac{T{\left(1-\rho \right)}^{2}}{\sigma^{2}}.$$

The Cramér–Rao lower bound (CRLB) is the asymptotic variance of the maximum likelihood estimator of *μ*. Because the off-diagonal elements of the FIM are zero, the CRLB is then given by the reciprocal of the right-hand side of equation (2.4):

2.5 $$\text{var}(\hat{\mu })=\frac{\sigma^{2}}{T{\left(1-\rho \right)}^{2}}.$$

We next derive the variance of the maximum likelihood estimator of *μ* when incorrectly assuming independent errors: $\epsilon (t)\overset{\mathrm{IID}}{∼}\;N(0,\sigma^{\prime })$. Under this *false* model, equation (2.5) indicates that the variance of maximum likelihood estimators is given by:

2.6 $$\text{var}(\tilde{\mu })=\frac{\sigma^{\prime 2}}{T}.$$

To meaningfully compare $\mathrm{var}(\tilde{\mu })$ with $\mathrm{var}(\hat{\mu })$, it is necessary to compare estimates of *σ*′, the standard deviation of noise for the false error model, with *σ*, the standard deviation of *ν*(*t*) in equation (2.2). To do so, we first compute the variance of the (true) AR(1) errors. This can be done by taking the variance of both sides of equation (2.2):

2.7 $$\text{var}(\epsilon (t))=\rho^{2}\text{var}(\epsilon (t-1))+\text{var}(\nu (t)).$$

Assuming the error process has a constant variance, equation (2.7) can be rearranged to yield:

2.8 $$\text{var}(\epsilon (t))=\frac{\sigma^{2}}{1-\rho^{2}}.$$

The false error model variance will broadly match the true process variance (otherwise there would be a mismatch between the width of the true and estimated error process) meaning *σ*′^2^ ≈ *σ*^2^/(1 − *ρ*^2^). By substituting this expression into equation (2.6) and comparing with equation (2.5), we see that true model parameter uncertainty exceeds that obtained from the false model, whenever

**Figure 2.**
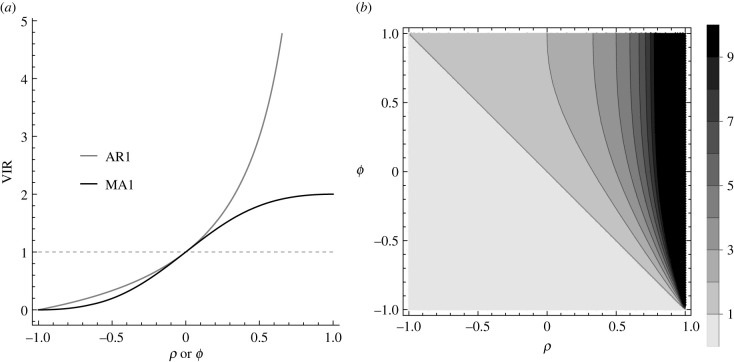
Variance inflation ratios for ARMA processes: (*a*) the VIR for AR(1) and MA(1) processes as a function of their respective parameters, and (*b*) the VIR for an ARMA(1,1) process.

2.9 $$\frac{\sigma^{2}}{T{\left(1-\rho \right)}^{2}}>\frac{\sigma^{2}}{T(1-\rho^{2})},$$

which is true when 0 < *ρ* < 1. The VIR is given by the ratio of the true error uncertainty to that estimated under the false model:

2.10 $$\begin{array}{rl} \text{VIR}(\rho ) & =\frac{1+\rho }{1-\rho }\\ & =1+\frac{2\rho }{1-\rho }, \end{array}$$

which is monotonically increasing with *ρ* throughout 0 < *ρ* < 1 (figure 2*a*), and lim _*ρ*→1_VIR(*ρ*) = ∞. Intuitively, as autocorrelation increases, each sample conveys less information about the underlying process, and parameter estimates have higher variance. Mischaracterizing data as independent, therefore, leads to overly precise estimates when the errors are positively autocorrelated.

In our experience, and through the results we present in §4, positive auto correlation (where *ρ* > 0) seems to more commonly occur. If negative autocorrelation does occur, equation (2.10) indicates that assuming independent noise will produce estimators with inflated variance, and, hence, VIR < 1 (figure 2*a*).

### 2.2. Nonlinear differential equation models

We now consider a model of the form:

2.11 x(t)=f (t;θ)+ϵ(t),

where, e.g. *f*(*t*; *θ*) is the solution of a nonlinear ODE (or a function of the solution of such an ODE) with univariate parameter *θ*. As mentioned earlier, the true error process is AR(1) as given by equation (2.2). In the electronic supplementary material, S1.1, we show that by the same logic as in §2.1, the VIR is given by:

2.12 $$\text{VIR}(\rho )=\frac{(1-\rho^{2})\sum_{t=1}^{T}{\left(\partial f/\partial \theta {|}_{t,\theta }\right)}^{2}}{\sum_{t=1}^{T}{\left(\partial f/\partial \theta {|}_{t,\theta }-\rho \partial f/\partial \theta {|}_{t-1,\theta }\right)}^{2}}.$$

If the differential equation solution is linear, its sensitivity is constant, i.e. ∂*f*/∂*θ* = const, and equation (2.10) for the constant mean model is recovered. If the differential equation has relatively weak nonlinearities, our simulations in §4 indicate that equation (2.10) nonetheless provides a reasonable approximation of equation (2.12).

If the model has multiple parameters, so that *θ* is a vector, it is possible to derive a VIR (see the electronic supplementary material, S1.2). However, this expression is not as straightforward to intuit as equation (2.12). Indeed, in some of our examples, it is not straightforward to calculate this quantity, and, instead, we approximate the VIR using equation (2.10).

Until this point, we have assumed that only the model parameters are unknown, but it is more typical that *σ*, *ρ* and/or the initial state of the system must also be estimated. The results in the electronic supplementary material, S1.3 and S1.4 show that, since the off-diagonal terms corresponding to *σ* and *ρ* are zero, that these parameters being unknown does not affect the variances of the *θ* estimates. In the electronic supplementary material, S1.5, we show that the off-diagonal terms corresponding to *θ* and the initial state of the system are generally non-zero: estimates of the model parameters can be correlated with the initial state estimates. This indicates that the exact VIR for model parameters is a less compact expression than equations (2.10) or (2.12) when the initial state is unknown. Our results in §4, however, indicate that equation (2.10) may nonetheless provide a reasonable approximation in some systems, even for substantially autocorrelated errors.

### 2.3. Moving-average processes

Our results thus far correspond only to AR(1) errors. Other types of autoregressive (AR) error processes also exist: one such class is the moving-average (MA) processes. In MA processes, the autocorrelation is generally less persistent than for AR processes. The simplest MA process is an MA(1) process, in which a measurement error is correlated with its value in the previous period, but not thereafter. This could occur if ephemeral, short-term factors influence consecutive measurements. An MA(1) process can be written as follows:

2.13 ϵ(t)=ν(t)+ϕν(t−1),

where $\nu (t)\overset{\mathrm{IID}}{∼}\;N(0,\sigma )$.

For simplicity of derivation, we revisit the ‘constant mean’ model described in §2.1, with errors described by an MA(1) process:

2.14 x(t)=μ+ν(t)+ϕν(t−1).

The steps involved in the calculation of the VIR for the MA(1) case mirror those involved for the AR(1) case and detailed calculations are given in the electronic supplementary material, S2. The VIR for the *μ* parameter of equation (2.14) is given by:

2.15 $$\text{VIR}(\mu )=1+\frac{2\phi }{1+\phi^{2}},$$

meaning the variance of the true model estimator exceeds the false model whenever *ϕ* > 0 and has a maximum value: VIR(*ϕ* = 1) = 2. In the electronic supplementary material, S2, we describe simulations which we performed to demonstrate the validity of equation (2.15). The electronic supplementary material, figure S1 shows the results of these and illustrates that empirical and theoretic VIRs are in good correspondence.

Figure 2*a* demonstrates that, whenever there is positive autocorrelation, VIR > 1, meaning that the estimator variance under the true noise model is greater than under the false model. In addition, if *ρ* = *ϕ* > 0 for each of an AR(1) and an MA(1) process, the VIR for the former always exceeds the latter. This makes intuitive sense since an AR(1) process has greater error persistence, meaning that the effects of model misspecification are amplified relative to the more transient MA(1) process.

### 2.4. Autoregressive moving-average noise processes

Noise processes may not neatly fall into either AR or MA processes, nor need they necessarily be of order 1. In general, noise may be a combination of these two processes, as in the following ARMA process formed by combining an AR(*p*) process with an MA(*q*) process (termed an ARMA(*p*, *q*) process):

2.16 $$\epsilon (t)=\rho_{1}\epsilon (t-1)+\cdots +\rho_{p}\epsilon (t-p)+\nu (t)+\phi_{1}\nu (t-1)+\cdots +\phi_{q}\nu (t-q).$$

These general processes can be rearranged using the lag operator, *L a*_*t*_ = *a*_*t*−1_ (see [18, ch. 2] for a discussion of the use and usefulness of lag operators) to:

2.17 $$\begin{array}{rl} \nu (t) & =\frac{1-\rho_{1}L-\cdots -\rho_{p}L^{p}}{1+\phi_{1}L+\cdots +\phi_{q}L^{q}}\epsilon (t)\\ & =\frac{\Psi_{p}(L)}{\Phi_{q}(L)}\epsilon (t), \end{array}$$

where $\Psi_{p}(L)$ and $\Phi_{q}(L)$ denote the corresponding lag operator polynomials. By using equation (2.17), we can determine the asymptotic variance of the maximum likelihood estimator for *μ* in the constant model defined by equation (2.1):

2.18 $$\text{var}(\hat{\mu })=\frac{\sigma^{2}}{T}\frac{\Phi_{q}{\left(1\right)}^{2}}{\Psi_{p}{\left(1\right)}^{2}}.$$

Equation (2.18) gives the variance of the maximum likelihood estimator of *μ* when assuming the correct error model. As mentioned earlier, we can also calculate the estimator variance when incorrectly assuming independent Gaussian noise. To do so requires that we calculate the variance of an ARMA(*p*, *q*) process, which for general *p* and *q* yields an unwieldy polynomial expansion. Instead, for illustration, we consider the ARMA(1,1) case, which has relatively simple variance [18] given by:

2.19 $$\text{var}(\epsilon (t))=\frac{1+\phi^{2}+2\phi \rho }{1-\rho^{2}}.$$

Thus, the VIR is given by,

2.20 $$\text{VIR}(\rho ,\phi )=\underset{\mathrm{VIR}\;\mathrm{of}\;\mathrm{AR}(1)}{\underbrace{\left(1+\frac{2\rho }{1-\rho }\right)}}\left(1+\frac{2\phi (1-\rho )}{1+\phi^{2}+2\phi \rho }\right),$$

which, as indicated, is the VIR for an AR(1) process multiplied by a factor. This factor exceeds 1 so long as *ϕ* > 0 and 0 < *ρ* < 1, meaning that the VIR for an ARMA(1,1) process exceeds the VIR for an AR(1) process (and, hence, also that of an MA(1) process) whenever there is positive autocorrelation in terms of both the autoregressive and moving-average terms of the error (see figure 2*b*). This makes intuitive sense since, if both constituents of an ARMA(1,1) process cause positive autocorrelation, the combined noise process has even greater autocorrelation.

In the electronic supplementary material, S3, we describe simulations to demonstrate the validity of equation (2.20). In the electronic supplementary material, figure S2, we show the results of these simulations which show that theoretical VIRs are in good correspondence with empirical values.

## 3. Applied modelling

In this section, we first describe in §3.1 approaches to diagnosing and modelling time series error processes. In §3.2, we then describe how to fit these models to data.

### 3.1. Determining an appropriate noise process

When analysing real data, it is generally not straightforward to know what type of measurement process to assume. The canonical assumption is that of IID normal measurements. After such a model is fitted, it is possible to test whether the errors—representing both measurement processes and model discrepancies—exhibit autocorrelation. Because the errors represent both of these factors, their autocorrelation does not necessarily reflect imperfections in the measurement process. However, if autocorrelation is detected, this forces the analyst to reflect on their chosen measurement model and potentially to refit their model using a more appropriate measurement process. This suggests the following workflow:

(i) use an optimizer to fit a model to data. This can be done by targeting either the maximum likelihood parameter values or, alternatively, the Bayesian maximum a posteriori (MAP) estimates. We denote the estimated parameter values by $\hat{\theta }$;
(ii) calculate the residuals: $\hat{\epsilon }(t)=x(t)-f\;(t;\hat{\theta })$. Note that these differ from the *true* errors ϵ(t) since they are obtained using the estimated parameter values rather than the true equivalents;
(iii) calculate the sample autocorrelation function: $\Gamma (\tau )=\mathrm{cor}(\hat{\epsilon }(t),\hat{\epsilon }(t-\tau ))$ for *τ* ∈ [1, 2, …, *τ*_max_]; and
(iv) if there is evidence of substantial autocorrelation, then consider whether this is owing to model misspecification or measurement processes. If the former, consider changing the underpinning mechanistic model. If the latter, do a refit assuming an autocorrelated noise model (this fit can either be done via maximization, for maximum likelihood estimation or MAP estimation; or using, e.g. a Markov chain Monte Carlo (MCMC) algorithm for a full Bayesian fitting).

**Figure 3.**
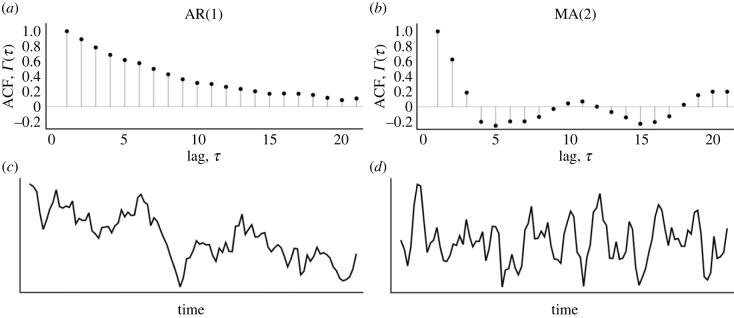
Autocorrelation functions for AR(1) and MA(2) series. (*c*,*d*) We show samples of length 100 of AR(1) and MA(2) processes, and (*a*,*b*) we show their respective sample autocorrelation functions. For the AR(1) process, we used *ρ* = 0.95 to generate the data; for the MA(2) process, we used *ρ* = 0.9 and *ϕ* = 0.8. ACF denotes the *autocorrelation function*.

However, if there is evidence of autocorrelated residuals, what autocorrelated noise model should be fitted? This depends on the problem at hand but can, as suggested earlier, be guided by the sample autocorrelation function of residuals obtained from fitting a model with independent Gaussian errors. For AR(1) processes, the autocorrelation function is [18]:

3.1 $$\Gamma (\tau )=\rho^{\tau },$$

i.e. when |*ρ*| < 1, an autocorrelation function that decays exponentially with lag (figure 3). For MA(1) processes, the autocorrelation function is:

3.2 $$\Gamma (\tau )=\left\{ \begin{array}{cc} \phi , & \text{if }\tau =1\\ 0, & \text{otherwise}. \end{array}\right.$$

So, for MA(1) processes, substantial autocorrelation occurs only at the first lag. More generally, for MA(*q*) processes, autocorrelation exists until the *q*th lag (figure 3). Indeed, whenever |*ρ*| < 1, it is possible to use the Koyck transformation to rewrite an AR(1) process as an MA(∞) process (with MA coefficients exactly mirroring the autocorrelations given in equation (3.1)), which provides some intuition for the interrelation between these two types of processes [18].

Choosing an ARMA error process that mirrors the autocorrelation patterns seen in the residuals provides a somewhat automated way of deciding on a noise model and, essentially, follows the approach forged by Box and Jenkins in their pathbreaking 1970s book (recent edition: [19]). This framework is, by no means, the only workflow followed, since applied time series modelling is, actually, a much broader church. An alternative popular approach falls under the banner of ‘structural time series (STS)’ or ‘state-space’ modelling, championed originally by Harvey for econometric time series [18]. In this philosophy, a time series is built up from various latent (i.e. not directly observed) components that represent characteristics of the series. For example, a series may be decomposed into stochastic time trends and cyclical components.

The STS approach is more model-driven and aims to decompose a series into understandable components. The STS framework is also naturally able to handle series that are non-stationary, where the probability distributions governing quantities like the mean and variance of the process vary over time. In the Box–Jenkins approach, by contrast, any non-stationarity is treated first by differencing the series, that is, via the operator, Δ_*s*_ *y*_*t*_ = *y*_*t*_ − *y*_*s*_, then by fitting an ARMA model to the transformed series—this combined process of differencing followed by fitting ARMA models is termed autoregressive integrated moving-average process (ARIMA) modelling.

Because both types of time series analysis—ARMA and STS—are used in practice, we do not suggest a single path here. In the two real data examples in §4, we initially follow Box–Jenkins and examine how well different ARMA models fit the residual series using the Akaike information criterion (AIC). This provides us with a guide as to whether models allowing autocorrelation better fit the data and hints as to which alternative models should be fitted—particularly as, in our examples, it is feasible that measurement apparatus imperfections could lead to residual autocorrelation.

### 3.2. Model fitting

When an appropriate error process has been chosen using the framework described in §3.1, it is necessary to fit the model to data. For ARMA processes, there are essentially two ways to fit such models to data: the first uses the generative process model to write down a conditional likelihood; the second, and more general approach, uses Kalman filters, which provide an efficient means to calculate likelihoods. An additional benefit of Kalman filters is that they can also handle STS-type models (see §3.1). Here, we describe how the first, and simpler, of these approaches can be used to fit an ODE model with ARMA(1,1) errors. The equivalent Kalman filter approach is provided in the electronic supplementary material, S4. In both cases, we suppose that the measurement equation for a univariate system observable is determined by the following system:

3.3 x(t)=f (t;θ)+ϵ(t) and ϵ(t)=ρϵ(t−1)+ν(t)+ϕν(t−1),}

where, as in previous cases, $\nu (t)\overset{\mathrm{IID}}{∼}\;N(0,\sigma )$.

To determine the likelihood for this model, we assume that the first two terms *ν*(1) = 0 and *ν*(2) = 0: this is known as a ‘conditional likelihood’ approach because we condition on initial values of processes.^1^ (Alternatively, rather than directly specifying *ν*(1) and *ν*(2), in a Bayesian framework, these can be set priors, allowing them to potentially take non-zero values.) For a given value of *θ*, the error can be directly calculated using ϵ(t)=x(t)−f (t;θ). Putting these together, we obtain:

3.4 ν(t)=ϵ(t)−ρϵ(t−1)−ϕν(t−1), ∀t>2.

Thus, the log-likelihood for this model is given by,

3.5 $$L=-\frac{T-2}{2}\log2\pi -\frac{T-2}{2}\log\sigma^{2}-\frac{1}{2\sigma^{2}}\sum_{t=3}^{T}\nu {\left(t\right)}^{2}.$$

The results shown in §4 of this article were generated assuming such a conditional likelihood approach.

## 4. Results

Here, we present results that illustrate the importance of assessing the validity of independent measurements and the consequences of failing to account for these measurement imperfections, when present. In §4.1, we first use synthetic data generated from a logistic model. In §4.2, we then use real data from cardiac electrophysiology experiments. In §4.3, we model outputs from electrochemistry experiments.

### 4.1. Logistic model

In this section, we use a simple ODE model to demonstrate how failing to account for autocorrelated measurements can lead to overly confident estimates; it also shows how mistakenly assuming independent measurements leads to more variable estimates. Here, we use the logistic model, which is a univariate ODE, with the solution determined from:

4.1 $$\frac{dx(t)}{dt}=rx(t)\left(1-\frac{x(t)}{\kappa }\right),$$

where *r* > 0 is a parameter determining the initial exponential growth rate, and *κ* = lim _*t*→∞_ *x*(*t*) is the carrying capacity; *x*(0) > 0 is the initial output value. The logistic model is common in mathematical biology, where it is typically used to describe resource-limited growth: imagine bacteria dividing on an agar plate—initially, bacteria have access to much resource, and the population density grows fast; later, once food becomes scarce, growth slows and the population eventually reaches a maximum size.

In our experiments, we generated *x*(*t*) using *r* = 0.5, *κ* = 50 and *x*(0) = 1. We then generated observations y(t)=x(t)+ϵ(t) and used AR(1) errors, ϵ(t), as described by equation (2.2), where we fixed *σ* = 1 and used five *ρ* values between 0.8 and 0.975 to generate synthetic datasets. For each *ρ* value, we generated a dataset consisting of 2000 equally spaced observations between *t* = 0 and *t* = 20. Ten such replicate datasets were generated for each *ρ* value. For each of these replicates, we fitted two statistical models: the correct one, which assumes AR(1) errors; the other, with IID Gaussian errors. For both models, we estimated *r*, *κ*, *x*(0) and *σ*; for the AR(1) model, we also estimated *ρ*. For the AR(1) model, we calculated the likelihood using the *generative model log-likelihood* approach described in §3.2. The priors that we use for each parameter are shown in the electronic supplementary material, table S1. The ODE was solved using Stan’s Runga-Kutta 4-5 solver [21]. These models are fitted using Stan’s NUTS MCMC algorithm [22] with 2000 iterations across each of four chains, with 1000 initial iterations discarded as warm-up. In all cases, $\hat{R}<1.01$ for all model parameters diagnosing MCMC convergence [23].

**Figure 4.**
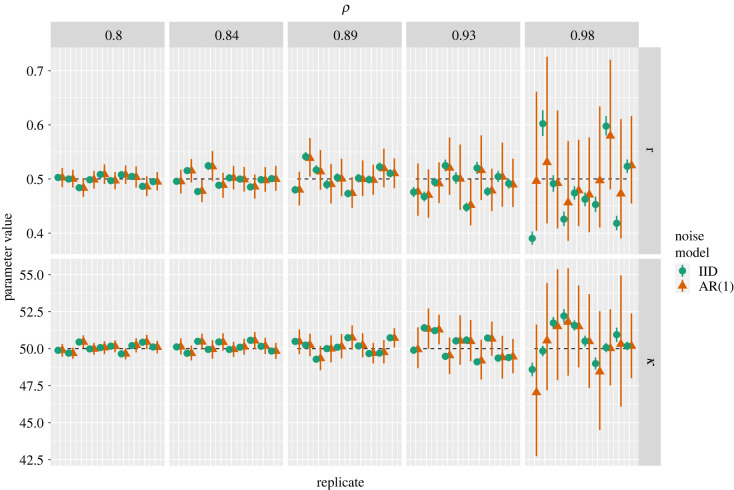
Logistic model: posteriors. Columns show results for each value of ρ=cor(ϵ(t),ϵ(t−1)) used to generate synthetic datasets as described in §4.1. Rows show posteriors for each of *r* and *κ*. Within each panel, we show posteriors from both the IID and AR(1) noise models; in each (of 10) replicates, both noise models were fitted to the same synthetic data which are shown as pairs of IID and AR(1) posteriors. Upper and lower whiskers represent 2.5% and 97.5% posterior quantiles; points represent posterior medians. Dashed lines show true parameter values.

In figure 4, we show summaries of the posterior distributions for the logistic model parameters for both the IID and AR(1) models fitted to each of the replicate datasets. The columns show results for different values of *ρ*; the rows show separate results for *r* and *κ* in equation (4.1). Within each panel, we show the IID and AR(1) posteriors for each replicate dataset.

We focus first on point estimates of the parameter values (the points and triangles in figure 4). Across the two model parameters and both noise models, the point estimates become more variable as *ρ* increases. Yet, over each set of replicates, the estimates appear relatively unbiased, with point estimates as likely to overestimate the true values as to understate them. The extent of variation, however, differs between the two models, and for 71% of replicates, the point estimate from the AR(1) model was closer to the true parameter value than the equivalent from the IID model.^2^ In the electronic supplementary material, figure S3, we quantify this by calculating the absolute percentage error in estimating each parameter value across all replicates at a given value of *ρ* for both noise models. This shows that the predictive errors for the logistic growth parameter, *r*, were between <1% and 21% over all *ρ* values considered; the errors for the carrying capacity, *κ*, were, in general, lower (range: <1%–6%). This difference in accuracy is probably owing to the somewhat narrower range of times when the model solution is sensitive to small changes in *r* as opposed to *κ*. The electronic supplementary material, figure S3 also shows that as *ρ* increases, both models get worse at estimating the true parameter; for *r*, the AR(1) model, however, does better on average than the IID one; for *κ*, both models perform similarly in terms of average error.

**Figure 5.**
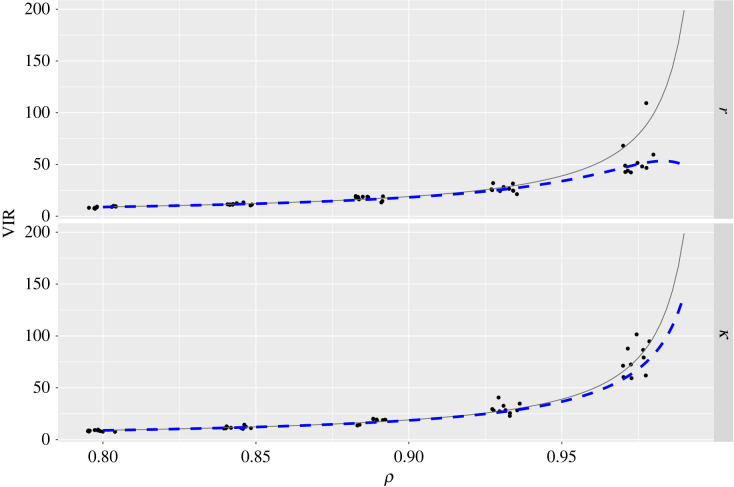
Logistic model: variance inflation ratios. The rows show results for the two logistic model parameters, *r* and *κ*. The vertical axis shows the estimated variance inflation ratios (VIRs) at each *ρ* value, which is the ratio of the AR(1) posterior variance to that of the IID model for each replicate (points). The lines show theoretically predicted VIRs: the blue-dashed line shows a more accurate VIR, accounting for uncertainty in the initial state (see the electronic supplementary material, S1.5), and the grey line plots equation (2.10) which ignores this uncertainty and treats the ODE solution as a linear model. Note, that horizontal jitter has been added to the points.

We next examine the uncertainty in estimates (the whiskers in figure 4). Across the two model parameters and both noise models, the posterior uncertainties widen as *ρ* increases. The extent to which they increase in width differs across both noise models, however, with the AR(1) uncertainties widening more acutely with changes in *ρ*. Indeed, for each replicate, we can calculate the ratio of the posterior variance for the AR(1) model to the IID model—in effect, estimating a VIR in each case—which we show in figure 5. The two rows here both show how the VIRs for each logistic model parameter increase along with *ρ*. To illustrate how our theory predicts this change, we also plot the theoretical VIR (blue-dashed lines; see the electronic supplementary material, S1.5) and the more approximate VIR which assumes the function is constant (equation (2.10); grey lines). Note that both VIRs plotted are somewhat approximate since they are derived from considering maximum likelihood estimates for an unbounded parameter, which is an approximation in this case since both *r* and *κ* are bounded below at zero, and we perform Bayesian inference using Gaussian priors. The theoretical results nonetheless capture well how the VIRs change with *ρ*, and equation (2.10) performs similarly to the more accurate result until the degree of autocorrelation is very high.

Finally, we examine how frequently the 95% posterior interval for the IID and AR(1) model posteriors encompass the true parameter value: we call these cases ‘successes’. In the electronic supplementary material, figure S4, we show the percentage of successes for *r* and *κ* at each value of *ρ* examined. Overall, this shows that the AR(1) posterior intervals more frequently encompass the true parameter value than the IID model. Indeed, across all values of *ρ* investigated, the maximum success percentage for *r* was 60% for the IID model and 100% for the AR(1) model (the results were qualitatively similar, albeit of different magnitudes for *κ*). In addition, as *ρ* increased, the frequency of success decreased for both parameters in the IID model; in all cases, the AR(1) model success frequencies did not change directionally with *ρ*.

Overall, our results show that using an inappropriate noise model results in more variable point estimates of parameters and uncertainties that are less reliable. This result has long been appreciated in time series regression analyses, where methods like generalized least squares—which essentially attempt to account for the structure of the noise—are commonly used when errors appear to deviate from IID Gaussian [17].

### 4.2. Cardiac electrophysiology model

We next examine a real dataset collected from experiments in cardiac electrophysiology on the *human Ether-à-go-go-Related Gene* (hERG) ion channel. These datasets are published in [24,25]. In the experiments, current from the hERG channel, which is often referred to as the rapid delayed rectifier potassium current, *I*_Kr_(*t*), is measured under a time-varying voltage stimulus *V*(*t*). The same laboratory experiment was conducted on five different cells, and we fit to each of these datasets separately, producing five sets of estimates.

Here, we model the current response of the hERG channel to this stimulus using an ODE model in the flavour of Hodgkin and Huxley’s (HH) landmark study [3]. This model contains two HH-style gating variables (‘activation’ *a* and ‘recovery’ from inactivation *r*) and a standard Ohmic expression:

4.2 $$I_{\mathrm{Kr}}(t)=g_{\mathrm{Kr}}\cdot a(t)\cdot r(t)\cdot (V(t)-E_{K}),$$

where *g*_Kr_ is the maximal conductance, and *E*_*K*_ is the reversal potential (Nernst potential) for potassium ions, which can be calculated directly from potassium concentrations using the Nernst equation. The voltage stimulus is a complicated ‘staircase-like’ function with no simple closed form: see [24] for further description. The gates *a* and *r* are governed by the ODEs:

dadt=a∞−aτa and drdt=r∞−rτr,a∞=k1k1+k2 and r∞=k4k3+k4 and τa=1k1+k2 and τr=1k3+k4,

where

k1=p1exp⁡(p2V) and k3=p5exp⁡(p6V)

and

k2=p3exp⁡(−p4V) and k4=p7exp⁡(−p8V).

The model has nine positive parameters to be inferred from the experimental data: maximal conductance *g*_Kr_ and kinetic parameters *p*_1_, *p*_2_, *p*_3_, …, *p*_8_. The initial conditions of the system were assumed to be: *a*(0) = 0 and *r*(0) = 1 and the system was solved for 100 s at *V* = −80 mV before running the staircase protocol.

Here, we assume that the measured current differs from the true current and is described by $I(t)=I_{\mathrm{Kr}}+\epsilon (t)$, where ϵ(t) is an error process that can either be IID Gaussian, $\epsilon (t)\overset{\mathrm{IID}}{∼}\;N(0,\sigma )$, or described by an AR process.

First, we use optimization to determine whether there is evidence of autocorrelation in the errors. To do so, we maximize the posterior assuming IID noise and from this to obtain a residual series. For optimization, we used CMA-ES [26], a derivative-free optimizer, as implemented in PINTS [27] following previous work [24,25]. In the electronic supplementary material, figure S5, we plot the sample autocorrelation function for the residuals for each of the cells, which illustrates strong and persistent autocorrelation, characteristic of AR processes. Across all cells, the estimated first-order residual autocorrelation was between 0.57 and 0.83.

We then compared the fit of the residual series to a range of ARMA processes: MA(1), AR(1) and ARMA(1,1), all of which could reasonably represent experimental artefacts: e.g. series resistance and leakage currents [28]. For each cell, we calculated the AIC for a range of ARMA(*p*, *q*) processes (where a lower AIC indicates a better fitting model [29]). The best ARMA model varied by cell and optimal *p* was between 1 and 5 and *q* from 2 to 5 (see the electronic supplementary material, figure S6). In the electronic supplementary material, figure S7, we show the result of these comparisons. Each panel of this figure corresponds to a cell. In each panel, we show the percentage difference between the AICs of each other process to the best fitting ARMA model (‘Min AIC’). In all cases, this shows that the IID Gaussian model is bettered by models encompassing autocorrelation. It also shows that the models incorporating AR terms outperformed the MA(1) model. In all cases, the ARMA(1,1) model produced a similar quality fit to the best model. Because of this, we decided only to attempt to perform Bayesian inference for the full model using the more parsimonious ARMA(1,1) noise compared to the best fitting ARMA(*p*, *q*) process.

To perform Bayesian inference, we used MCMC sampling for the IID, AR(1) and ARMA(1,1) noise models. For the sampling, we used population MCMC, which runs a series of chains at different ‘temperatures’ [30], using the default PINTS [27] algorithm settings. For each noise model and each of five cells, we ran four Markov chains with 150 000 iterations on each, with the first 50 000 of these discarded as warm-up; the draws were thinned by a factor of 10 after sampling.

While the ARMA(1,1) model was the best fit to the residuals, we could not achieve convergence with this model despite trying a range of informative priors on noise parameters. The difficulty of performing Bayesian inference for ARMA models has been noted before [31]. Because of this, we present results only for the IID and AR(1) models, which had $\hat{R}<1.1$ for all parameters. The priors specified for these two models are shown in the electronic supplementary material, table S2.

**Figure 6.**
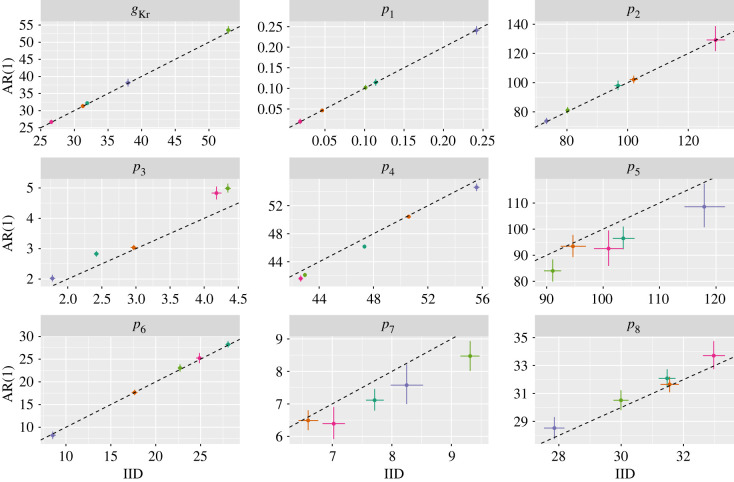
hERG model: posterior estimates. The horizontal axis shows the parameter values estimated from the IID noise model; the vertical axis shows the same for the AR(1) noise model. Points show posterior medians; whiskers indicate the 2.5% and 97.5% posterior quantiles. Colours indicate the experimental replicate (i.e. the cell on which experiments were performed). The dashed line shows the *y* = *x* line. Note, that the values for *g*_Kr_ have been scaled down by a factor of 1000; the *p*_3_ values have been scaled up by a factor of 100.

In figure 6, we compare the posterior distributions for the model parameters obtained across both noise models. For some parameters: *g*_*Kr*_, *p*_1_, *p*_2_ and *p*_6_, the estimates were similar across both the IID and AR(1) models; for others: *p*_3_, *p*_4_, *p*_5_, *p*_7_ and *p*_8_, there were often substantial differences. Despite these differences in parameter values, the IID and AR(1) models appeared visually to fit the data equally well (electronic supplementary material, figure S8). The extent to which the estimates differed also depended on the cell in question, with the cells shown in pink and dark green generally showing greater discrepancies.

To further investigate the cause of these discrepancies, in the electronic supplementary material, figure S9, we plot the posterior median *ρ* value from the AR(1) model versus the absolute percentage difference between the IID and AR(1) models. We also plot the best fit lines (in black) from linear regressions of the absolute difference on *ρ* for each parameter. Across all parameters, these indicate that as the magnitude of estimated error autocorrelation increased, there were greater differences between the IID and AR(1) model estimates.

**Figure 7.**
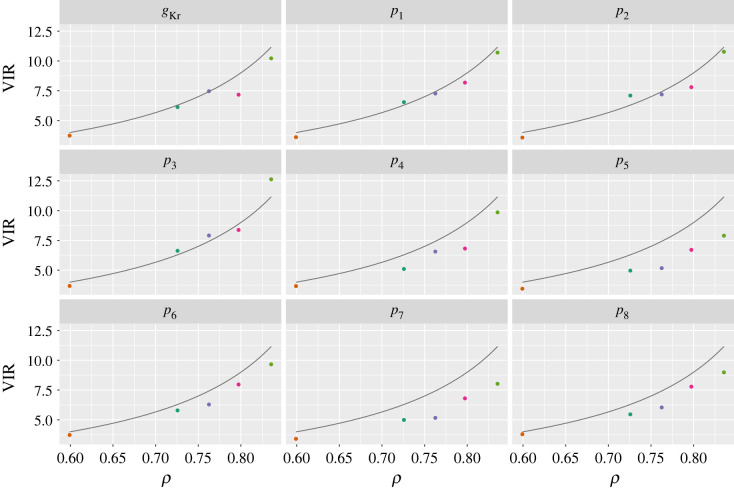
hERG model: VIRs. The horizontal axis shows the *ρ* posterior median values estimated from the AR(1) noise model; the vertical axis shows the estimated VIR for each parameter. Colours indicate the experimental replicate (i.e. the cell on which experiments were performed) and correspond with those shown in figure 6. The line shows the theoretical VIR for a linear model described by equation (2.10).

Finally, we estimate VIRs for each parameter across all cells in the system by taking the ratio of the AR(1) posterior variance to the IID equivalent. In figure 7, we plot these versus the estimated *ρ* value for each cell. In all cases, as *ρ* increased, the VIRs followed suit. In the same plot, we also overlay the theoretical VIR given by equation (2.10) for a linear model, since the nonlinear case is not straightforward to calculate for this model. Whilst the hERG model is nonlinear and the true noise process is unknown, in many cases, the theoretical VIR provided a reasonable guide as to how the variance increased with *ρ*.

### 4.3. Electrochemistry model

We next apply our methodology to a system in electrochemistry: unlike the previous examples, the model here is a partial differential equation (PDE), although yielding a single output—a current—which we fit to data. Since none of the theory derived in §2.2 assumes a particular form of the function, the results are not bespoke for ODEs. Because the PDE has only a single output time series, we use the same statistical framework as for our other examples. Further details are provided in the electronic supplementary material, S5.1.

In this example, we observed current time series, $\left\{{\tilde{I}}_{\mathrm{tot}}(t)\right\}$ resulting from a laboratory experiment. We assumed that ${\tilde{I}}_{\mathrm{tot}}(t)=I_{\mathrm{tot}}(t)+\epsilon (t)$, where ϵ(t) is an error process. We fixed a series of parameters in the model to experimentally determined values as given in the electronic supplementary material, table S3. On the remaining six parameters, we placed uniform priors as given in the electronic supplementary material, table S4.

**Figure 8.**
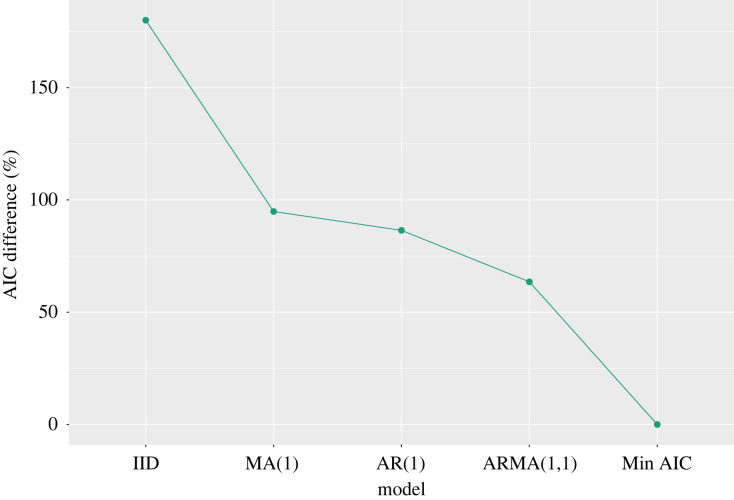
Electrochemistry model: AICs. The vertical axis shows the percentage difference between the AIC of each model (indicated on horizontal axis) compared to the best fitting model (‘Min AIC’).

To assess the level of autocorrelation in the error process, we follow the approach outlined in §3.1. In particular, we assumed that the noise process is IID Gaussian and used an optimizer, CMA-ES [26] (as implemented in PINTS [27]), to determine maximum likelihood estimates of the parameter values and to obtain a residual series. We then compared the fit of various ARIMA models to these residuals: in figure 8, we compare the AICs from IID, MA(1), AR(1) and ARMA(1,1) models to the one which minimized this criterion: an ARIMA(4,1,4) model. This shows that the IID Gaussian model is substantially bettered by models incorporating autocorrelation in the error series.

As part of this process, we also fitted to the residual series using various types of state-space models. To do this fitting, we relied on the *Statsmodels* Python package [32]. The state-space models we tried included a *local level* model, a *random walk with drift* model and a *random trend* model: all of these had substantially worse fits as determined by AIC compared to the ARIMA processes. Because of this, we did not go ahead with full Bayesian inference for these model types.

We next attempted to fit the electrochemistry model assuming AR(1), ARMA(1,1) and the ARIMA(4,1,4) error processes in a Bayesian model; we also fitted the model using a IID Gaussian error process for comparison. The models were fitted using the Haario–Bardenet adaptive-covariance MCMC algorithm in PINTS [27]. Uniform priors were set on all fitted parameters as described in [33]. The Markov chains were initialized to the MAP estimates found using the CMA-ES optimization algorithm. Three chains were run using 10 000 samples, the first 3000 of which were discarded as warm-up. Convergence was diagnosed via $\hat{R}<1.1$. We were unable to obtain Markov chain convergence for the ARIMA(4,1,4) model: we speculate that this was because the additional number of parameters of this model caused the inferred errors themselves to become unidentified.

**Figure 9.**
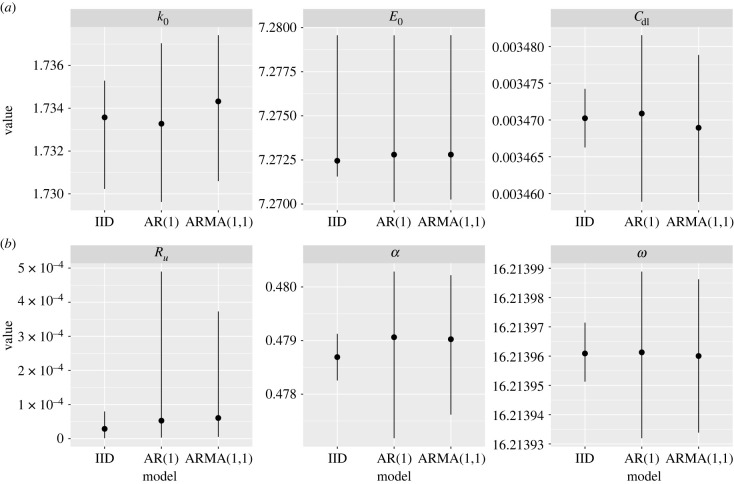
Electrochemistry model: posterior estimates. Posterior summaries for the IID, AR(1) and ARMA(1,1) models for each inferred parameter are shown. The points show the posterior median, and (*a*,*b*) show the 97.5% and 2.5% posterior quantiles, respectively.

In figure 9, we show the estimated posteriors for the IID, AR(1) and ARMA(1,1) models. In this figure, the panels show posterior summaries for each parameter across the three models. Across all parameters, the AR(1) and ARMA(1,1) models had increased uncertainty relative to the IID model. This was most notable for the uncompensated resistance *R*_*u*_, where the two models with autocorrelated errors produced distributions with longer tails. In addition, the median point estimates of parameters varied across the three models (again, most notably for *R*_*u*_).

## 5. Discussion

This work highlights how mischaracterizing the measurement process for ODE models can have marked consequences for inference. Our results indicate that failing to account for measurement-induced autocorrelation in errors results in overconfident estimates of parameter values, with the degree of overconfidence depending on the magnitude and type of stochastic process governing measurements. By using real data collected from experiments in cardiac electrophysiology and electrochemistry, we fit models assuming independent errors and obtained residual series that bore the hallmarks of autocorrelated errors. When these models were refitted assuming autocorrelated noise processes, we obtained considerably wider parameter bounds than when specifying independent noise. Whether this is a more general phenomena is unclear, but our results indicate that choice of measurement process can substantially affect inference. So choice of measurement process needs to be done with due care, and the types of diagnostic plots we use here can help to guide this process.

Misspecification of the ODE model can also generate autocorrelated errors, but its impact on inferences is probably different. When an ODE model is misspecified, parameter estimates (if these same parameters span both the correct and misspecified models) may display bias owing to parameter compensation [13,14]. Error autocorrelation owing to model misspecification could, in some cases, be modelled using some of the noise processes we describe here. Whether they should be, however, is less clear. It is possible that the two example systems we investigated did involve misspecified models and part of the observed autocorrelation was owing to this. We found that, by accounting for an autocorrelated error process, the uncertainty in the estimates was generally wider and, in some cases, the point estimates deviated considerably from the null IID Gaussian model. Because these are real life models, however, it is not straightforward to determine whether using an autocorrelated error model led to improved estimates. Future work, using toy models with known misspecifications and autocorrelated measurement processes could shed light on how best to account for both issues.

In this work, we considered a range of noise processes including ARMA and STS models. For our applied examples, sometimes complex autocorrelation structures were found to best fit the error variation, and it is questionnable whether measurement processes could have generated these errors. In addition, in some circumstances, the imposition of such measurement processes rendered the system practically unidentified, an issue with error processes which have long been recognized [31]. So how should an appropriate noise model be chosen? A noise process is itself a model, albeit a statistical one. Like other elements of the system, it should be understandable: if it is overly complex, the noise process is more likely to overfit current data resulting in poor generalization of the overall model. By contrast, when assuming independent noise, this can also often produce parameter sets that are more likely to overfit current data. We, hence, argue that using a low-order ARMA model or a relatively simple STS noise model is preferable in many circumstances by helping to guard against some of the larger effects of measurement model misspecification. We do not make rigid specifications as to the limiting complexity of these processes that are used but believe a reasonable litmus test is, ‘Could I convince a colleague that this noise process represents the actual measurement process?’ If the measurement process is well understood and arguments can be made for complex measurement processes, then this reasoning should be explicitly stated.

More mechanistic models of the measurement process may also lead to clearer understanding of the underlying biological processes. A recent study modelled the measurement process of patch-clamp experiments, accounting for series resistance, membrane and pipette capacitance, voltage offsets, imperfect compensations made by the amplifier and leak currents [28]. In explaining inter-cell variation through imperfections in measurement, this produced a more parsimonious explanation of the data than when assuming cell-specific ion current kinetics. Another study from parasitology examined laboratory experiments, where mosquitoes are infected with malaria parasites through membrane feeding assays [34]. By considering the measurement processes leading to observations—that experiments consist of mosquitoes being randomly sampled from a wider pool of specimens and each dissection representing an individual snapshot of the parasite dynamics—this resulted in novel estimates of key parameters in epidemiology.

Here, we considered only noise processes which had a fixed form over time, meaning our analysis does not consider either temporal or output-linked heteroscedasticity. Nor do the noise models we consider allow the autocorrelation structure itself to change with time. Recent work in related systems has shown that time-varying noise processes may provide a better representation, where, typically, throughout a time trace of an output variable, there are some regions of low autocorrelation and low variation punctuated by high autocorrelation/high uncertainty regions [35]. The general noise processes used to handle these temporal patterns are likely to be non-parametric and less amenable to direct analysis than the processes we consider here. However, our analytical results may nonetheless provide an approximate guide as to the impact on parameter inference of modelling noise using non-IID processes. We also did not consider measurement of multiple states of a system and the possible correlations across these, which, intuitively, should reduce the information content of observations. It has been empirically demonstrated that choosing the so-called *robust* error models, such as the Student-*t* and Huber distributions can lead to better estimates [11], and it is possible that the techniques we use here could produce useful analytical results when applied to those situations.

In systems where the state is measured repeatedly over short time intervals, such as those in electrochemistry, cardiac physiology and neuroscience, experimental limitations may mean that the assumption of independent measurements is suspect. In these types of systems, it may thus be better to assume an autocorrelated measurement model by default to mitigate against the risk of unrealistically precise estimates. As experimental methods are developed to allow collection of data at increasingly finer gradations, however, accounting for measurement imperfections will probably be increasingly important when performing inference.

## Data Availability

The experimental data used for the cardiac electrophysiology model section is openly available in previous publications: [24,25]. The electrochemistry data are available from: github.com/pints-team/electrochem_pde_model. The experimental methods for producing the data are detailed in [36]. More information about the various forms of data are also available in the electronic supplementary material [37].

## Endnotes

1 This approach follows the discussion in [20, ch. 5.6].

2 In experiments with more replicates (50 opposed to 10 per parameter set), we found qualitatively similar results, with 66% of AR(1) estimates closer to the true values than the IID equivalents (95% credible interval: 62%–70%, assuming a uniform prior.)
